# Environmental and family correlates of daily TV-watching time in children with autism spectrum disorder and typically developing children

**DOI:** 10.3389/fped.2026.1873394

**Published:** 2026-06-22

**Authors:** Preeta Rajandran, Sophia Ang, Woonyoung Song, Jaehoon Lee, Shin Ying Chu, Onn Wah Lee, Pui Juan Woi, Ling-Yi Lin, Chien-Ju Chang

**Affiliations:** 1 Faculty of Health Sciences, Centre for Healthy Ageing and Wellness (H-CARE), Speech Sciences Programme, Universiti Kebangsaan Malaysia, Jalan Raja Muda Abdul Aziz, Kuala Lumpur, Malaysia; 2 Department of Educational Psychology, Leadership, and Counseling, Texas Tech University, Lubbock, TX, United States; 3 Centre for Rehabilitation & Special Needs Studies, Faculty of Health Sciences, Universiti Kebangsaan Malaysia, Jalan Raja Muda Abdul Aziz, Kuala Lumpur, Malaysia; 4 Center for Community Health Studies (ReaCH), Faculty of Health Sciences, Universiti Kebangsaan Malaysia, Jalan Raja Muda Abdul Aziz, Kuala Lumpur, Malaysia; 5 Department of Occupational Therapy, College of Medicine, National Cheng Kung University, Tainan City, Taiwan; 6 Department of Child and Family Science, National Taiwan Normal University, Taipei, Taiwan

**Keywords:** autism spectrum disorder, children, family interaction, Malaysia, television watching

## Abstract

This study investigated environmental and family factors associated with daily television (TV) watching time among children with autism spectrum disorder (ASD) and typically developing (TD) children. The sample included 225 participants (65 ASD, 160 TD) aged 3 to 6 years. Data were obtained through caregiver-reported questionnaires assessing socioeconomic status (SES), indoor play spaces, family interaction during holidays, and daily electronic device usage time excluding television watching time. Regression analysis identified non-television electronic device usage time (*p* < .001) and indoor space availability (*p* = .012) as significant factors associated with TV watching time, while SES (*p* = .095) and family interaction during holidays (*p* = .072) demonstrated marginal associations. The final regression model demonstrated modest explanatory power (adjusted *R*² = .11). Children with ASD watched slightly less TV daily (*M* = 1.88, *SD* = 1.57 h) than TD children (*M* = 2.03, *SD* = 1.26 h). Greater family interaction during holidays was associated with lower TV watching time among children with ASD, suggesting a potential role of structured family engagement in supporting healthier TV watching habits. Lower SES and limited indoor play spaces were associated with longer TV watching time. These findings support existing WHO recommendations regarding limiting screen exposure in young children. This study highlights the potential importance of family involvement and environmental factors in shaping TV watching behaviours, particularly among children with ASD, and supports the need for targeted strategies to encourage healthier media habits.

## Highlights

- **Focus:** Investigated environmental and family correlates of daily TV-watching time in children aged 3-6 years with and without Autism Spectrum Disorder (ASD).
- **Key Findings:**
  - Lower socioeconomic status and absence of indoor play spaces are associated with increased TV watching time.
  - Higher family interaction time during holidays reduces TV watching time in children with ASD but not in typically developing children.
  - Daily electronic device usage significantly predicts longer TV watching time across groups.
- **Significance:**
  - Emphasizes the protective role of family engagement against excessive screen time, particularly in children with ASD.
  - Supports targeted interventions to promote healthier screen habits aligned with WHO guidelines.
  - Offers practical recommendations for clinicians, caregivers, and policymakers.

## Introduction

Parents increasingly use digital media as a convenient tool to calm or entertain their children, reflecting an increasing reliance on technology in modern parenting (1). The global COVID-19 pandemic further amplified this trend, with lockdowns and social restrictions implemented since 2019 contributing to a substantial rise in digital device usage among children (2, 3). This issue is especially pertinent for children with Autism Spectrum Disorder (ASD), who may be more susceptible to excessive screen exposure due to their unique developmental characteristics. While previous research has explored television (TV) watching habits among typically developing (TD) children, less is known about the factors associated with TV watching time among children with ASD. This study aimed to bridge this gap by investigating the environmental and family factors associated with daily TV watching time in both ASD and TD children. Understanding these factors may help inform targeted strategies to promote healthier TV watching habits and support optimal development in young children, particularly those with ASD.

Excessive television watching time has been associated with adverse cognitive, language, and social-emotional development outcomes in children (4, 5). The American Academy of Pediatrics, in a policy reaffirmed in 2022, discourages TV watching for children under two years old and recommends limiting total media exposure to no more than one hour daily for older children (34). A meta-analysis on screen time found that only 35.6% of children aged 2 to 5 years met this guideline (6). Although screen time encompasses multiple forms of media exposure, TV watching remains one of the most common sedentary screen-based activities among young children. Additionally, a longitudinal study involving 1,314 children aged 9 to 10 years revealed that each additional hour of television exposure at 29 months was associated with a 7% decrease in classroom engagement and a 6% reduction in mathematics achievement (7).

Previous studies have reported that children with ASD tend to demonstrate longer screen time than TD children (8). Mazurek and Wenstrup (9) found that children with ASD often exhibit a strong preference for screen-based activities due to their predictable, repetitive, and visually engaging nature, which may align with the cognitive strengths of this population. The time spent watching TV and playing video games among children with ASD has also been reported to be longer than that of their typically developing siblings. Moreover, children with ASD may experience more behavioural difficulties associated with excessive video game use (9). Investigating the relationship between developmental status and TV watching time may provide valuable insights into how developmental differences relate to media viewing behaviours. Including TD children as a comparison group may also help clarify whether factors associated with TV watching differ according to developmental status.

When examining the relationship between developmental status and TV watching time, it is important to consider various factors including socioeconomic status (SES), environmental influences, and family dynamics. Previous studies have consistently reported associations between SES and children's screen-based viewing behaviours. Most studies found that TD children from low-SES families tend to demonstrate longer screen time (10, 11). However, further investigation is needed to determine whether SES demonstrates similar associations with TV watching time among both children with ASD and TD children.

Environmental factors, such as the availability of play spaces, have also been associated with lower TV watching time among TD children (8, 12, 13). These studies suggest that access to indoor and outdoor play spaces may encourage physical and social activities, which could contribute to lower screen exposure. However, it remains unclear whether play space availability demonstrates similar associations with TV watching time among children with ASD, as they may engage in more solitary activities, including TV watching (14, 15). Understanding how children with ASD perceive stimulation from play activities compared to TV watching could help clinicians provide more targeted recommendations for families.

The complex interplay between family dynamics and screen-related behaviours presents unique challenges for researchers and practitioners working with both TD children and children with ASD. Previous studies have shown that family interaction through shared activities has been associated with lower screen usage among TD children by providing meaningful social engagement and alternative forms of entertainment (16). Staiano et al. (16) also reported that family interactions during holidays were associated with lower screen time. In contrast, families of children with ASD may face additional challenges during family interactions, which could limit opportunities for engagement and be associated with greater reliance on screens (17, 18).

Although previous studies have explored screen-related behaviours among children, limited research has examined how environmental and family-related factors are associated specifically with TV watching time among children with ASD compared to TD peers. In particular, the combined influence of socioeconomic status, play space availability, and family interaction on TV watching behaviours remains insufficiently understood in the ASD population. Therefore, this study aimed to identify environmental and family factors associated with daily TV watching time among children with ASD and TD children. Understanding these associations may help inform family-centred approaches to promote healthier TV watching habits in early childhood.

## Methods

### Participants

Participant demographic characteristics are presented in Table 1. The study sample consisted of 225 caregivers of children aged between 3 years and 6 years 11 months, including 65 caregivers of children with ASD (29%) and 160 caregivers of TD children (71%). Participants were recruited through special education centres, community organizations, and online platforms in Malaysia.

**Table 1:** Demographic characteristics of participants.

Variable	All *n* (%)/Mean (*SD*)	ASD *n* (%)/Mean (*SD*)	TD *n* (%)/Mean (*SD*)
Age (years)	4.46 (1.07)	4.63 (1.03)	4.38 (1.08)
Sex			
Male	121 (56.8%)	43 (69.4%)	78 (51.7%)
Female	92 (43.2%)	19 (30.6%)	73 (48.3%)
Socioeconomic status (SES)			
High	72 (33.0%)	21 (32.8%)	51 (33.1%)
Low	146 (67.0%)	43 (67.2%)	103 (66.9%)
Indoor space availability			
Yes	204 (90.7%)	53 (81.5%)	151 (94.4%)
No	21 (9.3%)	12 (18.5%)	9 (5.6%)
Outdoor space availability			
Yes	188 (83.6%)	51 (78.5%)	137 (85.6%)
No	37 (16.4%)	14 (21.5%)	23 (14.4%)
Living status			
Both parents	177 (78.7%)	50 (76.9%)	127 (79.4%)
Father only	13 (5.8%)	6 (9.2%)	7 (4.4%)
Mother only	18 (8.0%)	5 (7.7%)	13 (8.1%)
Other	17 (7.6%)	4 (6.2%)	13 (8.1%)
Sibling presence			
Yes	163 (72.4%)	42 (64.6%)	121 (75.6%)
No	62 (27.6%)	23 (35.4%)	39 (24.4%)
Daily TV watching time (hr)	1.99 (1.35)	1.88 (1.57)	2.03 (1.26)
Non-television electronic device usage time (hr)	1.64 (1.32)	1.66 (1.47)	1.62 (1.25)
Family interaction time during holidays (hr)	2.90 (1.68)	2.42 (1.64)	3.10 (1.66)

Percentages for ASD and TD groups were calculated based on subgroup totals for available responses. Variations in subgroup totals reflect missing data for selected demographic variables.

The inclusion criteria required caregiver report that the child had received a prior clinical diagnosis of ASD from healthcare professionals based on DSM-5 criteria, while TD children were identified based on caregiver report indicating the absence of developmental, neurological, or diagnosed behavioural conditions. Children with significant medical conditions or severe intellectual disabilities that could substantially affect daily functioning or media-related behaviours were excluded from the study.

### Data collection

Data were collected using a close-ended caregiver questionnaire adapted from the KIT (Kids in Taiwan: National Longitudinal Study of Child Development and Care) project (2016) developed by the National Taiwan Normal University (NTNU). The KIT project was developed by a multidisciplinary team of researchers in pediatric healthcare, child development, early childhood education, family science, and statistical analysis to monitor child development and growing environments in Taiwan (19). The questionnaire, originally designed for children aged 36–72 months, was available in both Mandarin and English, and permission for its use in this study was obtained from the research team. Several relevant items were extracted, with minor modifications made to adapt cultural terminology to the Malaysian context.

Respondents were required to read the participant information sheet and complete the consent and demographic forms before answering the questionnaire. The questionnaire assessed children's average daily TV watching time and non-television electronic device usage time (e.g., smartphones, tablets, and computers), socioeconomic status (SES), access to indoor and outdoor play spaces, and family interaction time during holidays.

SES was determined based on caregiver-reported household income categorized according to Malaysian household income classifications. Low SES referred to households earning up to RM5,249 per month, while high SES referred to households earning above RM11,819 per month. Indoor and outdoor play space availability referred to caregiver-reported access to safe and usable play areas within or outside the home environment. Family interaction time during holidays referred to the average amount of time spent engaging in shared family activities during weekends or public holidays. Variables were measured using a combination of dichotomous and ordinal categorical items.

### Data analysis

Best-subsets analysis was conducted to identify optimal predictors associated with children's daily TV watching time. While regression analysis is commonly used to assess theoretical relationships or develop predictive models, the aim of this study was to identify a subset of variables that best explained variation in children's TV watching time. The best-subsets approach compares multiple regression models based on summary statistics, allowing model selection according to goodness-of-fit, parsimony, and theoretical relevance. This method was selected instead of traditional stepwise regression because it allows researchers to evaluate the predictive performance of alternative models simultaneously (20).

In this study, the dependent variable was daily TV watching time, which was square-root transformed to improve normality. Independent variables included socioeconomic status (SES) based on household income category (high or low), availability of indoor space (yes or no), availability of outdoor space (yes or no), living status (both parents, father only, mother only, or others), sibling presence (yes or no), disability status (ASD or TD), family interaction time during holidays, and non-television electronic device usage time. Interactions involving disability status were also examined.

A total of 2,441 regression models containing combinations of main effects and interaction effects were generated and fitted using ordinary least squares (*OLS*) regression. The final model was selected based on Akaike Information Criterion (*AIC*) and adjusted *R²* values (21). Regression analyses were conducted using R software (22), while descriptive analyses were conducted using IBM SPSS Statistics Version 26.0.

Statistical significance was set at *p* < .05. Findings with *p*-values between.05 and.10 were interpreted cautiously as marginal associations.

## Results

On average, children watched TV for 1.99 h (*SD* = 1.37) per day, with slightly lower TV watching time observed among children with ASD (*M* = 1.88, *SD* = 1.57) compared to TD children (*M* = 2.03, *SD* = 1.26). The best-subsets analysis identified a four-predictor model with one interaction term as the optimal model for explaining children's daily TV watching time (*AIC* = 379.32, adjusted *R*² = .11). The final model included SES, indoor space availability, non-television electronic device usage time, family interaction time during holidays, disability status, and the interaction between disability status and family interaction time during holidays. Table 2 presents the regression results for the final model. As the model was fitted using square-root transformed daily TV watching time, caution is required when interpreting coefficient values directly.

**Table 2:** OLS regression results of final model.

Parameter	*b*	*SE*	*t*	*p*	95% CI	*η*²p
(Intercept)	2.07	0.17	12.36	**<**.**001**	[1.76, 2.43]	-
Non-television electronic device usage time	0.06	0.01	4.09	**<**.**001**	[0.02, 0.10]	.073
SES: High	−0.14	0.08	−1.68	.095	[−0.28, 0.03]	.013
Indoor space: No	0.34	0.13	2.53	.**012**	[0.05, 0.62]	.029
Disability status: TD	−0.13	0.18	−0.68	.495	[−0.47, 0.23]	.002
Family interaction time during holidays	−0.05	0.02	−2.07	.**040**	[−0.08, 0.00]	.020
Disability status × Family interaction time during holidays	0.05	0.03	1.81	.072	[0.00, 0.09]	.015

95% confidence intervals (CI) were computed from 1,000 bootstrapping samples.

Bold values indicate statistically significant results (*p* < .05).

Non-television electronic device usage time demonstrated a statistically significant association with TV watching time (*p* < .001), with children watching approximately an additional 0.16 h (9.6 min) of TV for each additional hour of electronic device usage. SES demonstrated a marginal association with TV watching time (*p* = .095), with children from low-SES households tending to watch more TV compared to children from high-SES households. Indoor space availability was significantly associated with lower TV watching time (*p* = .012). In addition, the interaction between disability status and family interaction time during holidays demonstrated a marginal association with TV watching time (*p* = .072).

**Figure 1:**
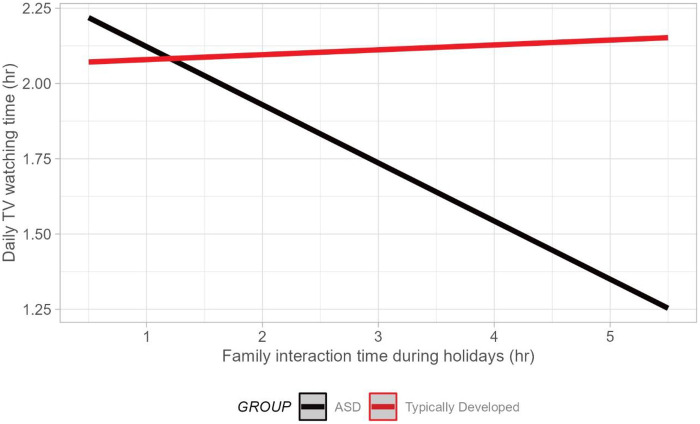
Interaction between disability Status and family interaction time during holidays.

Figure 1 illustrates the estimated TV watching time among children with ASD and TD children across varying levels of family interaction time during holidays. Family interaction time appeared to have minimal association with TV watching time among TD children. In contrast, children with ASD appeared to demonstrate lower TV watching time as family interaction time increased during holidays, although this interaction should be interpreted cautiously due to its marginal statistical significance.

## Discussion

The findings of this study suggest that non-television electronic device usage time was associated with longer TV watching time among young children, while children with ASD demonstrated slightly lower TV watching time compared to TD children. This finding differs from several previous studies reporting greater screen exposure among children with ASD (8). One possible explanation is that many children with ASD in the present study were recruited from child development or intervention centres, where parents may have received guidance regarding screen-related behaviours and media management. In addition, the present study specifically examined TV watching time rather than overall screen exposure, which may partially explain differences from previous literature examining broader screen-related behaviours.

Although the association between increased electronic device usage and longer TV watching time is noteworthy, potential confounding factors such as socioeconomic status, parental education, and family dynamics should also be considered. Previous studies have associated excessive screen exposure with poorer sleep quality (23), attention difficulties (24), and delayed language development (4, 25), particularly among young children (26). Future interventions may benefit from addressing overall screen exposure across multiple devices while encouraging balanced media use and age-appropriate content. However, the feasibility and effectiveness of such approaches should be interpreted within the context of cultural differences, technological accessibility, family routines, and broader social influences.

This study also identified several environmental and family-related factors associated with children's TV watching time, including socioeconomic status (SES), indoor space availability, and family interaction during holidays. Children from lower-SES households tended to demonstrate longer TV watching time, consistent with previous studies examining screen-related behaviours among young children (10, 11, 27, 28). This pattern may reflect reduced access to alternative recreational opportunities, structured activities, or engaging play environments within lower-SES households. In addition, access to indoor play spaces was significantly associated with lower TV watching time, suggesting that engaging physical environments within the home may support reduced sedentary media behaviours among young children.

These findings may have practical implications for clinicians and families by highlighting the potential importance of environmental opportunities and family routines in shaping children's TV watching behaviours. Strategies that encourage accessible play opportunities and family-based engagement may help support healthier TV watching habits, although further longitudinal research is needed to better understand these relationships.

The interaction between TV watching time and family interaction time during holidays provided additional insight, despite the absence of statistically significant group differences in TV watching time between children with ASD and TD children. Family interaction time appeared to have minimal association with TV watching time among TD children. In contrast, children with ASD appeared to demonstrate lower TV watching time as family interaction during holidays increased, although this interaction demonstrated only marginal statistical significance and should therefore be interpreted cautiously.

Previous studies have suggested that children with ASD may be more drawn to screen-based activities because of their structured and predictable characteristics (9). The findings of the present study suggest that increased family engagement during holidays may potentially serve as an alternative source of social and recreational engagement for children with ASD. This observation is consistent with Dong et al. (29), who reported shorter screen time among children with ASD during weekends compared to weekdays, potentially due to increased parent-child interaction during non-working days.

Improved parent-child interaction has also been associated with healthier psychosocial outcomes and lower screen exposure among children (30). In addition to potentially limiting TV watching opportunities, parent-child interaction may support cognitive (31), social, and emotional development (32, 33). Therefore, family-centred approaches that encourage shared activities during holidays or weekends may be beneficial in supporting healthier TV watching habits among children with ASD.

Several explanations may account for this observed pattern. Increased family interaction time may provide children with ASD alternative sources of engagement beyond TV watching. Structured family activities may also help establish routines and boundaries surrounding TV watching behaviours. Furthermore, positive parent-child interactions may help fulfil social and emotional needs that could otherwise contribute to greater reliance on screen-based activities. Nevertheless, these interpretations remain speculative due to the cross-sectional nature of the study, and future longitudinal research is necessary to further examine these relationships.

In this context, it is also important to consider both global and local recommendations regarding screen exposure. According to the World Health Organization (35), children under the age of two years should not be exposed to screen activities, while children aged two to four years should be limited to no more than one hour of screen time daily. In Malaysia, the Malaysian Dietary Guidelines for Children and Adolescents (36) recommend limiting children's daily screen exposure to two hours, although specific age-based recommendations are not provided. These recommendations highlight the importance of supporting healthier media-related habits among young children, particularly those with developmental vulnerabilities such as ASD.

## Limitations

Several limitations should be considered when interpreting the findings of this study. First, participants in the ASD group were primarily recruited from child development centres where they were receiving intervention services. As the questionnaire captured TV watching behaviours only within the previous three months, the study did not account for potential differences in behaviours before or after intervention exposure. Parents who received education regarding media management during intervention programs may have modified their child's TV watching habits, which could have influenced the findings. Future studies may benefit from collecting longitudinal data across different stages of intervention to better understand changes in TV watching behaviours over time.

Second, the study relied on caregiver-reported measures of TV watching time and electronic device usage, which may be subject to recall bias. Future research should consider incorporating objective measurement approaches, such as digital tracking tools or device monitoring systems, to improve the accuracy of media exposure assessment.

In addition, the present study did not differentiate between different types of screen content, such as educational and non-educational media. Variations in content type may influence developmental outcomes differently, and future studies should further examine how specific forms of media content relate to children's cognitive, social, and behavioural development.

Finally, the cross-sectional nature of the study limits conclusions regarding causal relationships between family interaction, environmental factors, and TV watching time. Future longitudinal studies are needed to provide a more comprehensive understanding of how these variables may relate to changes in children's media viewing behaviours over time.

## Conclusion

This study highlighted the multifaceted environmental and family factors associated with children's daily TV watching time, including non-television electronic device usage, socioeconomic status, indoor space availability, and family interaction. The findings suggest that family involvement may play an important role in shaping TV watching behaviours, particularly among children with ASD. Future studies should focus on developing and evaluating interventions that address environmental and familial factors to promote healthier TV watching habits and support positive developmental outcomes among young children.

## Data Availability

The raw data supporting the conclusions of this article will be made available by the authors, without undue reservation.
